# When brain implants go mobile: rethinking neural probe design for dynamics and intelligence

**DOI:** 10.1093/nsr/nwaf455

**Published:** 2025-10-21

**Authors:** Kairu Dong, Qi Chen, Kewang Nan, Enming Song, John A Rogers

**Affiliations:** State Key Laboratory of Advanced Drug Delivery and Release Systems, School of Pharmacy, Zhejiang University, China; Institute of Optoelectronics & College of Future Information Technology, Institute of Science and Technology for Brain-Inspired Intelligence, Fudan University, China; State Key Laboratory of Advanced Drug Delivery and Release Systems, School of Pharmacy, Zhejiang University, China; Institute of Optoelectronics & College of Future Information Technology, Institute of Science and Technology for Brain-Inspired Intelligence, Fudan University, China; Shanghai Frontiers Science Research Base of Intelligent Optoelectronics and Perception, Fudan University, China; Querrey Simpson Institute for Bioelectronics, Northwestern University, USA; Department of Biomedical Engineering, Northwestern University, USA; Department of Materials Science and Engineering, Northwestern University, USA; Department of Mechanical Engineering, Northwestern University, USA

Long-term implantable bioelectronic systems, serving as human–machine interfaces or advanced surgical tools, offer a powerful means for direct information transmission between biological tissues and external computers, for innovative applications such as muscle–exoskeleton integration, neuromodulation and chronic disease management [1].

For minimally invasive implantation and chronic operation, the delivery of such bioelectronic systems should demand only small surgical incisions to minimize tissue damage and reduce infections. These requirements are most effectively addressed with devices that assume linear geometric shapes. Currently, however, conventional metal electrode microwires have diameters of ∼30 μm and do not readily support multi-electrode interfaces. Flexible filaments that support patterned thin films of metals support multi-electrode interfaces, but suffer from limitations in interconnect routing. An alternative approach involves the rolling of thin films patterned in similar ways to produce 3D scrolls [2]. For example, Liu *et al.* reported a high-density 1024-channel probe for brain-wide recordings formed by wrapping a microelectrode array (MEA) film onto a tungsten wire (from 101.3 ± 5.6 to 175.6 ± 8.2 μm) [3]. In another scheme, Guan *et al.* reported the self-assembly of the mesh section of an ultra-flexible MEA into a tubular structure with an average diameter of 220.1 ± 14.6 μm [4].

A key limitation of existing MEAs is their inability to dynamically interact with the body. As static implants, these structures cannot be adjusted or repositioned easily [5]. Moreover, most implantable bioelectronic systems focus on electrophysiology, with few examples of multimodal sensing operation. A goal, therefore, is to develop multifunctional bioelectronic devices, in mobile formats that are stable, conformable and, ultimately, intelligent under remote control as smart implants.

**Figure 1.**
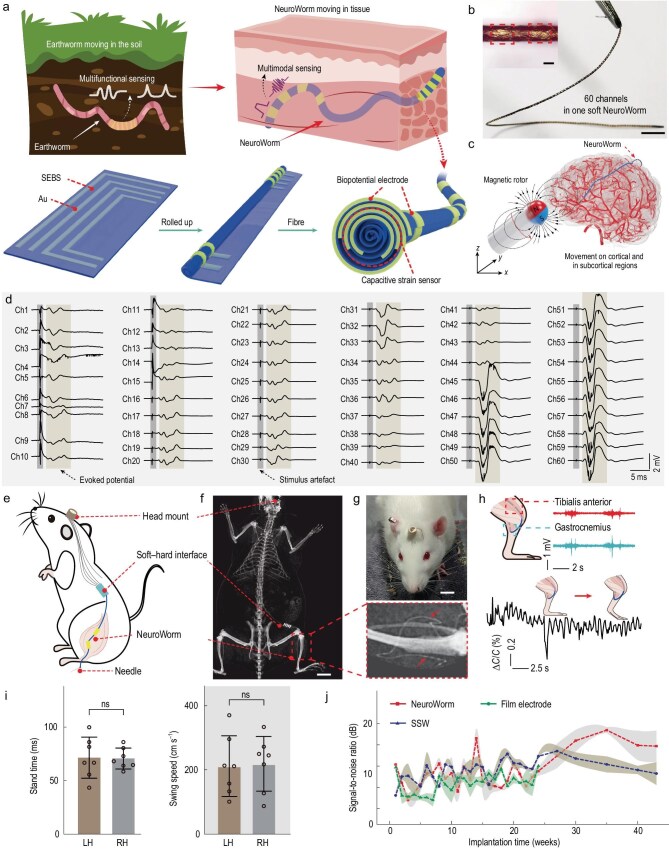
Movable, implantable multimodal fiber platform for long-term *in vivo* biointerfacing. (a) Schematic diagram of the design and fabrication strategy of the bioelectronics named NeuroWorm. (b) Typical fabricated microfiber with 60 biopotential electrodes longitudinally distributed along its length. Scale bars, 1 cm (main), 100 μm (inset). (c) Illustration of the NeuroWorm movement in the brain under magnetic field control. (d) Evoked biopotential signals monitored simultaneously from a single 60-channel fiber sensor in an acute experiment. (e, f) Schematic and X-ray image of the NeuroWorm system implanted in the hindlimb muscle of a rat. Scale bar, 2 cm. (g) Photograph of a rat with the implanted NeuroWorm. Scale bar, 1 cm. (h) Simultaneous electromyography recording from two muscle sites and strain signal acquisition during leg extension. (i) Gait analysis 10 days post-implantation in the right tibialis anterior. Left hindlimb indicates the contralateral (non-implanted) limb and right hindlimb denotes the implanted side. Stand time (contact-to-off time) and swing speed were analysed by using a two-tailed paired *t*-test (*n* = 3 independent rats). Data are presented as mean ± SD; ns, not significant (*P* = 0.8934 for stand time, *P* = 0.8529 for swing speed). (j) Longitudinal comparison of signal-to-noise ratio for NeuroWorm, SSW and thin-film electrodes over 43 weeks post-implantation. Data are presented as mean ± SD (*n* = 3; three signal segments were analysed at different time points for each electrode type).

Recently, an interesting article published by Xie *et al.* [6] proposed a dynamic platform with some of these characteristics, which they refer to as a NeuroWorm—a movable long-term implantable soft microfiber, as shown in Fig. 1. The authors use a 400-nm-thick styrene ethylene butylene styrene substrate, onto which gold conductive wires are patterned via vacuum thermal evaporation through shadow masks. Rolling of the film forms a 3D electrode array, self-encapsulated and with exposed electrode sites as tissue interfaces. As described, the process allows the integration of ≤60 discrete channels along a single fiber, with a minimum diameter of ∼109 μm. Additionally, a miniature magnetic bead incorporated at the tip allows external magnetic steering.

The NeuroWorm offers capabilities in both electrophysiological and biomechanical monitoring. From the standpoint of mechanics, the fiber is stretchable to strains of 93 ± 3.2%, with a Young’s modulus of 3.1 ± 0.2 MPa and stable conductivity under 30% strain over 1000 cycles. Electrically, multichannel signal recordings exhibit minimal crosstalk and stable impedance (∼1 MΩ at 1 kHz after 28 weeks *in vivo*). In rats, this device stably acquired electromyography signals for >43 weeks, with a signal-to-noise ratio superior to that possible with conventional rigid electrodes. Furthermore, an integrated capacitive strain sensor detected mechanical strains as low as 0.1% with a gauge factor of 0.96 and excellent linearity (*R*^2^ = 0.9998). These capabilities enable high-quality, long-term, multimodal biosignal acquisition in dynamic tissue environments.

*In vivo* experiments in rabbits evaluated the performance of NeuroWorm for brain interfaces. Under real-time digital subtraction angiography imaging, the soft microfiber was magnetically guided through cortical and subcortical regions via a minimal 5-mm cranial opening, with positioning controlled by a 700-mT external magnetic field. The navigation process proceeded smoothly without damage to the device or significant trauma to the tissue. Along the 109-μm-diameter fiber, the integrated 60-channel electrode array consistently recorded electrocorticography and local field potential signals with stable amplitude and minimal noise during magnetic manipulation. Maintaining signal quality during movement suggests reliable electrode–tissue coupling and adequate mechanical compliance.

These proof-of-concept demonstrations of this dynamic bioelectronic system highlight ultra-flexible multimodal microfiber and post-implantation magnetic steering. With the ingeniously structured layout of the gold films and the frontier fabrication process, the movable implantable soft fiber realized hybrid sensing of electrophysiological and biomechanical signals, showing minimal signal crosstalk and damaged tissue. These results represent an important technological advance and conceptual breakthrough toward next-generation fiber-based active and intelligent bioelectronics in the context of long-term, minimally invasive and mobile evaluation of the nervous system.
